# Comparative Effects of Verapamil, Nicardipine, and Nitroglycerin on Myocardial Ischemia/Reperfusion Injury

**DOI:** 10.1155/2011/521084

**Published:** 2011-03-02

**Authors:** Hitoshi Yui, Uno Imaizumi, Hisashi Beppu, Mitsuhiro Ito, Munetaka Furuya, Hirofumi Arisaka, Kazu-Ichi Yoshida

**Affiliations:** Division of Anesthesiology, Department of Clinical Care Medicine, Kanagawa Dental College, 82 Inaokacho, Yokosuka-shi, Kanagawa 238-8580, Japan

## Abstract

The aim of this experiment was to establish whether verapamil, nicardipine, and nitroglycerin have (1) infarct size-limiting effects and (2) antiarrhythmic effects in *in vivo* rabbit hearts during ischemia/reperfusion. Rabbits received regional ischemia by 30 min of left anterior descending coronary artery occlusion followed by 3 hours of reperfusion under ketamine and xylazine anesthesia. The animals were randomly assigned to the following 4 treatment groups: a control group, a verapamil group, a nicardipine group, and a nitroglycerin group. A continuous infusion of verapamil, nicardipine, or nitroglycerin was initiated 5 min prior to ischemia. Infarct size/area at risk decreased in verapamil, and nitroglycerin. The incidence of ischemia-induced arrhythmia decreased in nicardipine, verapamil and nitroglycerin. The incidence of reperfusion-induced arrhythmias decreased in verapamil and nitroglycerin. From the present experimental results, verapamil and nitroglycerin rather than nicardipine did afford significant protection to the heart subjected to ischemia and reperfusion in a rabbit model.

## 1. Introduction

Myocardial ischemia/reperfusion injury and its prevention have become the focus of considerable attention. It was reported that intracellular calcium overload is a recognized common pathway that can explain ischemia/reperfusion injury [1]. 

Several studies have shown that *L*-type calcium antagonists have beneficial effects on the ischemic myocardium by inhibiting transmembrane calcium influx in cardiac and vascular smooth muscle [2, 3].

On the other hand, previous experimental studies have investigated effects of nitric oxide (NO) donors to reduce myocardial ischemia/reperfusion injury [4, 5]. 

 This cardioprotection is due to the mechanism that nitroglycerin is converted in vascular smooth muscle cells to NO and relaxes vascular muscle of the large veins and coronary arteries. As a result, nitroglycerin can reduce preload and also provide an increased blood flow to the heart. 

It is clear that calcium antagonists and NO donors have cardioprotective effects. However, there is no study examining the intensity of myocardial protection of these drugs as indexes of infarct size and arrhythmias. 

 The aim of this experiment was to establish whether verapamil, nicardipine, and nitroglycerin have infarct size-limiting effects and antiarrhythmic effects in *in vivo* rabbit hearts during ischemia/reperfusion.

## 2. Materials and Methods

The present study was performed in accordance with the Guidelines of Animal Care and Use Committee of Kanagawa Dental College.

### 2.1. General Surgical Preparation

Male New Zealand White rabbits weighing 2.7–3.2 kg were allowed *ad libitum* access to standard laboratory stock diet and water. Animals were initially anesthetized with ketamine (35 mg·kg^−1^) and xylazine (5 mg·kg^−1^) given intramuscularly. Five mL of 1% lidocaine was subcutaneously injected as an additional local anesthetic during the initial surgical procedures. Tracheotomy was performed and rabbits were intubated with an uncuffed endotracheal tube (ID 3.5 mm). The animals were ventilated with room air supplemented with additional oxygen using mechanical ventilator (Shinano, SN-480-5, Tokyo, Japan) and a semiclosed breathing circuit (Shinano, SN-487, Tokyo, Japan). End-tidal CO_2_ partial pressures were continuously monitored using a multigas monitor (Datex, Capnomac, Helsinki, Finland). Ventilator rate was 30–35 breaths per minute and tidal volume was between 30–35 mL. The respiratory rate was frequently adjusted to maintain P_a_O_2_ greater than 100 mmHg, P_a_CO_2_ at 35–45 mmHg, pH 7.35–7.45, and Base Excess between −3 and +3. After the left jugular vein was exposed and cannulated with a polyethylene catheter, 0.9% sodium chloride (0.15 mL·min^−1^) was continually administered during the experiments. The carotid artery was dissected out and fluid-filled polyethylene tube was placed in it and connected immediately to an electrocardiogram monitor (Nihon-kohden Co, Life scope 11, Tokyo, Japan) *via* pressure transducer (Nihon-kohden Co, TP-400T, Tokyo, Japan) for arterial pressure recording. An electrocardiogram was recorded throughout the experiment *via* lead II of the standard electrocardiogram. Ischemia or reperfusion-induced arrhythmias included premature ventricular contraction (PVC) and ventricular tachycardia (VT). After all the surgical procedures had been performed, a 15-minute period was allowed for stabilization. Anesthesia was maintained with ketamine and xylazine solution (ketamine 35 mg·kg ^−1^·h^−1^, xylazine 5 mg·kg ^−1^·h^−1^ i.m.; K/X) with room air supplemented with additional pure oxygen. 

 Left thoracotomy was performed and pericardium was opened to expose the heart. A silk thread ( K-890H, Ethicon, Somerville, NJ ) with taper C-1 needle was passed around the left anterior descending artery (LAD) and the end of the tie was threaded through a small vinyl tube to form a snare. The LAD was occluded by pulling the snare, which was then fixed by clamping the tube with a mosquito hemostat. The rabbits were given 500 units of heparin for preventing thrombus formation in the coronary artery after reperfusion. Myocardial ischemia was confirmed by regional cyanosis, ST segment elevation, and decreased blood pressure. Reperfusion was confirmed by reactive hyperemia over the surface after releasing the snare.

### 2.2. Study Groups and Experimental Protocol

**Figure 1:**
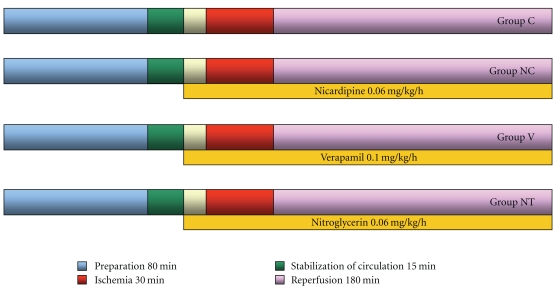
Schematic diagram of the protocol. Group C: a control group (*n* = 9). Group NC: a nicardipine group (*n* = 5). Group V: a verapamil group (*n* = 8). Group NT: a nitroglycerin group (*n* = 10).

The experimental design used in the current study is illustrated in Figure 1. Rabbits received regional ischemia by 30 min of the LAD occlusion followed by 3 hrs of reperfusion under ketamine/xylazine (K/X) anesthesia. Before this, rabbits were randomly assigned to the following 4 treatment groups (*n* = 5 ~ 9, resp.): a control group (Group C), verapamil (Group V), nicardipine (Group NC), and nitroglycerin (Group NT) treatment group. A continuous infusion of verapamil (0.1 mg·kg^−1^·h^−1^), nicardipine (0.06 mg·kg^−1^·h^−1^), or nitroglycerin (0.06 mg·kg^−1^·h^−1^) was initiated 5 min prior to ischemia. Therapeutic dose of each drug was administered.

#### 2.2.1. Determination of Area at Risk and Infarct Size

Following completion of experimental protocol, the *in vivo* visualization of the myocardium at risk was accomplished with reocclusion of the coronary artery and injection of 10% Evans blue into the venous cannula until the eyes turned blue. The Evans blue was allowed to circulate for about 30 sec to demarcate the risk and nonrisk regions. The hearts were quickly excised under deep anesthesia with 5% sevoflurane and frozen. The frozen hearts were then cut into six transverse slices of equal thickness. The area at risk was determined by negative staining with Evans blue. The slices were stained by incubation for 15 min in 1% triphenyl tetrazolium chloride (TTC) in isotonic pH 7.4 phosphate buffer. After staining, the sections were placed in formalin for preservation, and measurements of risk area, infarct area, and left ventricle were made with computer-aided morphometry. From each section, the ischemic risk area (unstained by blue dye) and the infarcted area (unstained by TTC) were outlined and measured by planimetry. The area from each region was averaged from the slices. Infarct size was expressed as a percentage of the area at risk.

### 2.3. Hemodynamic Measurements

Hemodynamic measurements included systolic, diastolic, mean arterial blood pressures, and heart rate. Rate pressure product was calculated as the product of heart rate and peak arterial pressure. Baseline hemodynamic measurements were taken prior to any experimental manipulations. Subsequently, the measurements were taken at preischemia, 30 min of ischemia, and 30 and 60 min of reperfusion.

### 2.4. Limitation Section

Rabbits which could not be survived until the end of experiments with lethal arrhythmias such as ventricular fibrillation (VF) have been excluded from the data analysis and not considered further.

### 2.5. Statistical Analysis

Between-group differences in area at risk/left ventricle, infarct size/area at risk, and duration of arrhythmias were assessed by Kruskal-Wallis test followed by Dunn's procedure as a multiple comparison procedure. 

Statistical comparisons of individual hemodynamic parameters between groups were made by using one-way analysis of variance (ANOVA) followed by Fisher's protected least significant difference. Bartlett's test for equality of variances was used to ensure the validity of statistical comparison using the one-way ANOVA.

The difference in the percentage incidence of arrhythmias was analyzed with a *χ* ^2^ test. 

All data are reported as group mean ± SD and were considered statistically significant at a probability value (*P*) less than  .05.

## 3. Results

Hemodynamics during ischemia and reperfusion are shown in Table 1. Heart rate (HR) was significantly reduced in Group NT 30 min after reperfusion. Mean arterial blood pressure (MAP) was significantly reduced in Group V and Group NT at any point measured and in Group NC 60 min after reperfusion and preischemia. Rate pressure product (RPP) did not alter significantly among Group C, Group NC, and Group V. However, there was significant difference between Group C and Group NT 30 min after ischemia, and 30 and 60 min after reperfusion. 

**Figure 2:**
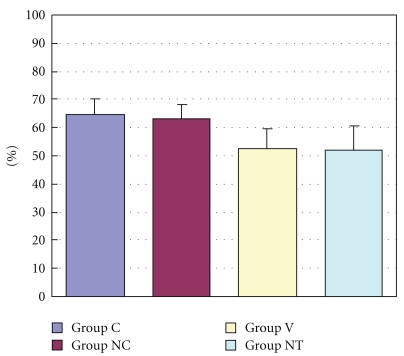
The figure shows R/L value (%), which means the area at risk expressed as percentage of left ventricle. Data are expressed as mean ± S.D. Area at risk revealed no significant difference among all groups, suggesting that the changes in the infarct size observed among the groups did not depend on R/L. Group C: a control group. Group NC: a nicardipine group. Group V: a verapamil group. Group NT: a nitroglycerin group.

**Figure 3:**
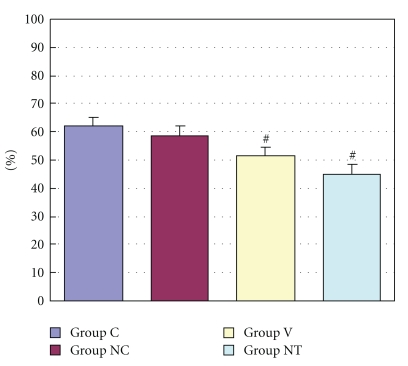
The figure shows I/R value (%), which means the infarct size expressed as percentage of area at risk. Group C: a control group. Group NC: a nicardipine group. Group V: a verapamil group. Group NT: a nitroglycerin group. # Significantly different (*P* < .05) from Group C (mean ± S.D.).

The ratio of areas at risk (*R*) to left ventricle (*L*) ranged from 51.9 ± 8.7% to 64.8 ± 5.3% with no significant difference among all the groups (Figure 2).  Figure 3 shows the infarct size expressed as percentage of area at risk in four groups. Mean infarct size was 62.1 ± 3.1% of *R* in Group C and decreased significantly in Group V (51.3 ± 3.1%) and Group NT (45.1 ± 3.6%) (*P* < .05 versus Control) (Figure 3). Compared with Group C, myocardial protective effect was observed in the Group V and Group NT. 

**Figure 4:**
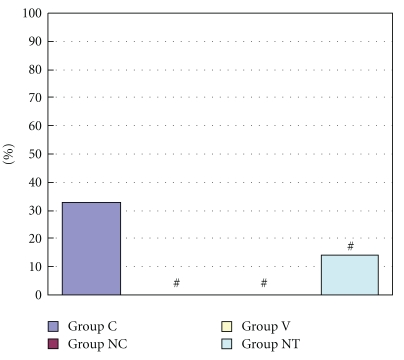
The figure shows incidence of arrhythmias during myocardial ischemia. Group C: a control group. Group NC: a nicardipine group. Group V: a verapamil group. Group NT: a nitroglycerin group. # Significantly different (*P* < .05) from Group C (mean ± S.D.).

**Figure 5:**
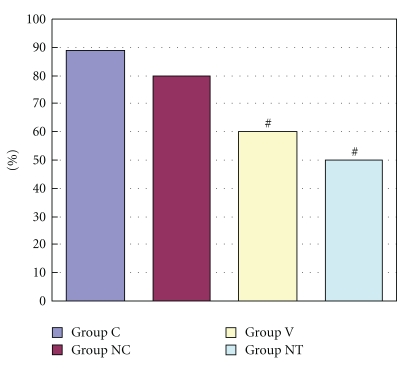
The figure shows incidence of arrhythmias during reperfusion. Group C: a control group. Group NC: a nicardipine group. Group V: a verapamil group. Group NT: a nitroglycerin group. # Significantly different (*P* < .05) from Group C (mean ± S.D.).

**Figure 6:**
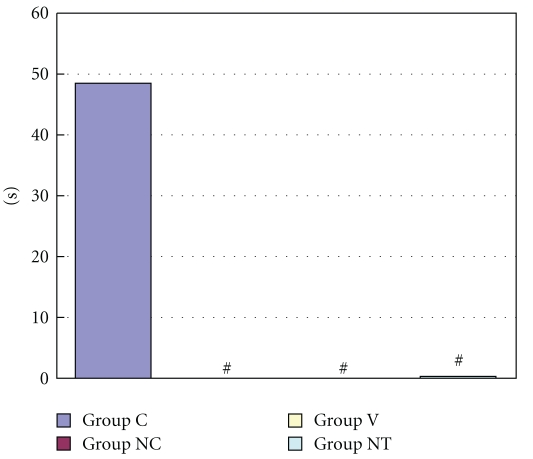
The figure shows duration of arrhythmias during myocardial ischemia. Group C: a control group. Group NC: a nicardipine group. Group V: a verapamil group. Group NT: a nitroglycerin group. # Significantly different (*P* < .05) from Group C (mean ± S.D.).

**Figure 7:**
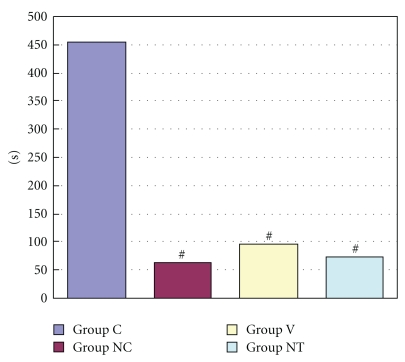
The figure shows duration of arrhythmias during reperfusion. Group C: a control group. Group NC: a nicardipine group. Group V: a verapamil group. Group NT: a nitroglycerin group. # Significantly different (*P* < .05) from Group C (mean ± S.D.)

 The incidence of arrhythmias during myocardial ischemia was 33.3% in Group C, 0% in Group NC and Group V, and 14.3% in Group NT (Figure 4). The incidence of arrhythmias during reperfusion was 88.9%, 80.0%, 60.0%, and 50.0% in Group C, Group NC, Group V, and Group NT, respectively (Figure 5). Duration of arrhythmias during myocardial ischemia were 48.4 sec, 0 sec, 0 sec, and 0.2 sec in Group C, Group NC, Group V, and Group NT, respectively (Figure 6). Duration of arrhythmias during reperfusion were 453.7 sec, 63.8 sec, 97.1 sec, and 73.0 sec in Group C, Group NC, Group V, and Group NT, respectively (Figure 7).

## 4. Discussion

The present study shows that verapamil and nitroglycerin rather than nicardipine did afford significant protection to the heart subjected to ischemia and reperfusion in a rabbit model as indexes of myocardial infarct size and duration of ventricular arrhythmias during ischemia and reperfusion.

 Protocol to prevent necrosis by calcium-blocker may vary greatly among researchers with regard to the choice of drugs, dose, time of administration, collateral blood flow of the animals, and difference of experiment system [2, 6]. In this experiment, we used rabbit hearts of which coronary collateral blood flow is almost zero like human hearts [7, 8]. 

Reimer and Jennings reported that verapamil reduced infarct size induced by 40 min of ischemia in *in vivo* dog, but failed to limit infarct size when the period of ischemia was prolonged to 3 hours. However, pretreatment with verapamil 15 min before ischemia prevented necrosis of myocytes, though the treatment started 15 min after the onset of ischemia failed to limit infarct size [2]. The ability of verapamil to reduce infarct size seems to be affected by the duration of ischemia and time of initiation of therapy. In this study, pretreatment with verapamil 5 min before ischemia reduced infarct size induced by 30 min of ischemia. It seems that pretreatment with verapamil before ischemia may be required to reduce infarct size in rabbit hearts.

*L*-type calcium antagonists such as verapamil have positive effects on contractile function of ischemic myocytes during ischemia and have improved postischemic recovery of function [9]. The rational for using verapamil to reduce myocardial infarct size includes their ability to (1) reduce oxygen demand by reducing afterload, preload and contractility; (2) enhance oxygen supply to the ischemic zone by relieving coronary vasospasm and vasocontraction, (3) prevent ischemia-induced calcium overload; (4) preserve mitochondrial structure and function [9, 10], (5) decrease the availability of calcium to stimulate ATPase, proteases and lipases [6]. Our study showed that the rate pressure product, which is one index of myocardial oxygen demand, did not significantly differ between the control and verapamil groups. This suggests that the infarct size-limiting effect by verapamil does not depend on myocardial oxygen demand and supply.

 On the contrary, nicardipine failed to decrease infarct size. Nicardipine is a dihydropyridine derivative and a potent coronary and cerebral vasodilator [3]. Although in baboons and dogs, infarct size-limiting effects of nicardipine have been previously reported [3, 11], nicardipine had no appreciable activity in our rabbit model. It is probably due to the differences of receptivity of nicardipine, species, administration, length of ischemia, and extent of collateral blood flow [7, 8]. Verapamil mainly affects the myocardium, whereas nicardipine exerts a greater effect on smooth muscle in the peripheral vasculature. It may cause reflex tachycardia if peripheral vasodilatation is marked, resulting in a substantial decrease in blood pressure [12]. It is assumed that nicardipine-induced tachycardia, unlike verapamil, might increase myocardial oxygen consumption and reduce the infarct size-limiting effect [13]. However, it does not seem to be this mechanism because nicardipine did not significantly affect HR and RPP in this study.

Nitroglycerin has been used to treat angina and heart failure. It is assumed that nitroglycerin is converted in vascular smooth muscle cells to NO or an NO congener (S-nitrosothiol, SNO), which activates guanylate cyclase and thus relaxes vascular smooth muscle [14]. Their vasodilator activity decreases myocardial oxygen demand by reducing venous return to the heart. NO, therefore, plays a crucial role in cardioprotection [15]. At therapeutic doses, nitroglycerin has two major effects. First, it causes dilation of the large veins, resulting in pooling of blood in the veins. This diminishes preload and reduces the work of the heart. Second, nitroglycerin dilates the coronary vasculature, providing an increased blood supply to the heart muscle. Nitroglycerin decreases myocardial oxygen consumption because of decreased cardiac work [12]. Our experimental data also demonstrated that nitroglycerin reduced RPP. Therefore, smaller myocardial infarct size may be explained by smaller oxygen consumption. 

Nitroglycerin has been shown, in both human and animal studies, to induce a prospective phenotype that limits tissue damage after ischemia and reperfusion. This phenomenon is similar to ischemic preconditioning, and several reports suggest that the molecular pathways involved in this protective effect of nitrates are the same that determine ischemic preconditioning [16]. 

 A previous investigation from our laboratory demonstrated that pretreatment with nitroglycerin 30 min before ischemia failed to limit infarct size [17]. However, the same dose of nitroglycerin reduced infarct size when it was administered 5 min before ischemia. This indicates that the ability of nitroglycerin to reduce infarct size depends on the timing of its administration. 

 Myocardial ischemia/reperfusion injury might significantly impair heart functions and induce arrhythmias via cellular Ca^2+^ overload [18]. Generally, the main cause of ischemia-induced arrhythmias is aberrations in impulse generation or a defect in impulse conduction [12] which is prevented from calcium-blocker. On the other hand, the main cause of reperfusion-induced arrhythmias remains poorly studied. A delay after depolarization-induced activity and an increase in Ca^2+^ concentration are thought to play a role. Blocking the *L*-type Ca^2+^ channel has been shown to be effective in preventing reperfusion arrhythmias in rats [18, 19]. In this study, verapamil, nicardipine, and nitroglycerin have antiarrhythmic effects during myocardial ischemia. It was suggested that verapamil and nicardipine could reduce arrhythmias by decreasing influx of *L*-type calcium current. Only nicardipine did not decrease incidence of arrhythmias during reperfusion. Nicardipine, used to treat hypertension, exerts a stronger effect on the vascular muscle than on the heart [12]. Therefore, it is not clinically used as antiarrhythmic drug. As for nitroglycerin, there is a report that NO, which is converted from nitroglycerin, blocks *L*-type calcium channels [20]. There is a possibility that nitroglycerin reduced incidence and duration of ventricular arrhythmia by blocking calcium channel.

## 5. Conclusions

In this investigation, verapamil and nitroglycerin decreased myocardial infarct size and incidence and duration of ventricular arrhythmias during ischemia and reperfusion. According to the present experimental results, verapamil and nitroglycerin rather than nicardipine did afford significant protection to the heart subjected to ischemia and reperfusion in a rabbit model.

## Figures and Tables

**Table 1:** Hemodynamics during ischemia and reperfusion.

	Group	Preischemia	30 min after ischemia	30 min after reperfusion	60 min after reperfusion
HR (beats/min)	Group C	158.1 ± 18.9	171.7 ± 12.8	163.9 ± 14.9	159.9 ± 14.7
	Group NC	168.0 ± 25.7	153.0 ± 10.8	154.6 ± 18.9	164.8 ± 32.5
	Group V	167.3 ± 22.6	176.4 ± 27.1	169.3 ± 19.3	162.0 ± 24.8
	Group NT	155.1 ± 19.0	156.9 ± 20.2	146.3 ± 19.9*	147.6 ± 12.1

MAP (mmHg)	Group C	70.0 ± 5.1	63.0 ± 8.0	63.8 ± 7.9	64.3 ± 8.8
	Group NC	49.8 ± 13.0^#^	55.8 ± 8.5	58.4 ± 8.7	51.5 ± 11.5^#^
	Group V	56.8 ± 7.2^#^	52.4 ± 5.5^#^	51.4 ± 4.4^#^	51.3 ± 4.6^#^
	Group NT	52.5 ± 5.5^#^	52.8 ± 3.6^#^	52.7 ± 4.2^#^	52.5 ± 2.1^#^

RPP (mmHg/min)	Group C	13167.2 ± 562.0	12765.5 ± 606.6	12478.4 ± 552.1	12438.1 ± 563.2
	Group NC	11201.8 ± 1459.6	11316.8 ± 1233.0	11387.4 ± 1219.1	11318.6 ± 1418.4
	Group V	11421.3 ± 417.0	11270.3 ± 548.1	10918.9 ± 430.1	10485.0 ± 469.0
	Group NT	10799.0 ± 348.7	10584.2 ± 239.2^#^	10002.5 ± 445.0^#^	9863.7 ± 362.0^#^

HR: heart rate. MAP: mean arterial blood pressure. RPP: rate pressure product. ^#^Significantly different (*P* < .05) from Group C (mean ± S.D.). *Significantly different (*P* < .05) from Group V (mean ± S.D.).
